# Identification of the cytoplasmic DNA-Sensing cGAS-STING pathway-mediated gene signatures and molecular subtypes in prostate cancer

**DOI:** 10.1186/s12885-024-12492-3

**Published:** 2024-06-14

**Authors:** Jie Yang, Zihan Xu, Weitao Zheng, Yifan Li, Qiang Wei, Lu Yang

**Affiliations:** 1 Present Address: Department of Urology, West China Hospital of Sichuan University, Sichuan Province, Chengdu, China; 2 China Agricultural University, Beijing, 100083 China

**Keywords:** cGAS-STING pathway, Prostate cancer, Biochemical recurrence, Molecular subtype, Gene signature

## Abstract

**Background:**

Considering the age relevance of prostate cancer (PCa) and the involvement of the cGAS-STING pathway in aging and cancer, we aim to classify PCa into distinct molecular subtypes and identify key genes from the novel perspective of the cGAS-STING pathway. It is of significance to guide personalized intervention of cancer-targeting therapy based on genetic evidence.

**Methods:**

The 430 patients with PCa from the TCGA database were included. We integrated 29 key genes involved in cGAS-STING pathway and analyzed differentially expressed genes and biochemical recurrence (BCR)-free survival-related genes. The assessments of tumor stemness and heterogeneity and tumor microenvironment (TME) were conducted to reveal potential mechanisms.

**Results:**

PCa patients were classified into two distinct subtypes using AURKB, TREX1, and STAT6, and subtype 1 had a worse prognosis than subtype 2 (HR: 21.19, *p* < 0.001). The findings were validated in the MSKCC2010 cohort. Among subtype 1 and subtype 2, the top ten mutation genes were MUC5B, DNAH9, SLC5A10, ZNF462, USP31, SIPA1L3, PLEC, HRAS, MYOM1, and ITGB6. Gene set variation analysis revealed a high enrichment of the E2F target in subtype 1, and gene set enrichment analysis showed significant enrichment of base excision repair, cell cycle, and DNA replication in subtype 1. TME evaluation indicated that subtype 1 had a significantly higher level of T cells follicular helper and a lower level of plasma cells than subtype 2.

**Conclusions:**

The molecular subtypes mediated by the cGAS-STING pathway and the genetic risk score may aid in identifying potentially high-risk PCa patients who may benefit from pharmacologic therapies targeting the cGAS-STING pathway.

## Introduction

Prostate cancer (PCa) is the most common malignancy among males in about 60% country worldwide, which is the second most frequent cancer and the fifth leading cause of cancer death among males [1]. The precise pathogenesis of PCa remains uncertain, however, it is acknowledged that PCa is a heterogeneous age-related ailment, and its occurrence exhibits a quadratic rise with advancing age [2–4]. This observation implies that aging might exert a conceivably pivotal function in the initiation and/or progression of PCa. Aging denotes the outcome of time-dependent aggregation of cellular impairments accompanied by a gradual decline in physiological integrity, resulting in compromised functionality. Furthermore, the cumulative cellular damage during aging may sporadically confer anomalous benefits upon specific cells, ultimately instigating carcinogenesis [5]. Deoxyribonucleic acid (DNA) damage accumulation represents a shared feature of the aging process and assumes a causal function in various premature aging disorders [6, 7]. Despite the existence of an intricate network of DNA repair mechanisms aimed at addressing a considerable portion of nuclear DNA damage induced by external and internal stresses [8], the repair capacity is limited and the unrepaired DNA damage will persist and accumulate with age, eventually leading to aging and death. In addition, the integrity of mitochondrial DNA (mtDNA) is an important part of the genomic stability systems, most mtDNA mutations caused by replication errors occur in the origin of life and increase by polyclonal expansion in aging. The accumulated mtDNA mutations damage respiratory chain dysfunction, leading to mitochondrial dysfunction in different tissues[9]. The progressive deterioration of mitochondrial function during the aging process leads to apoptosis, resulting in the release of mtDNA into the cytoplasm via BAX/BAK macropores or voltage-dependent anion channel (VDAC) pores. Additionally, the mitochondrial permeability transition pore (MPTP) may serve as an alternative pathway for inner mitochondrial membrane traversal of mtDNA, enabling its release from mitochondria to the cytoplasm [10]. Cancer cells frequently exhibit an abundance of cytoplasmic double-stranded DNA (dsDNA) derived from various sources, including the genome, mitochondria, and exogenous origins. Nevertheless, chromosomal instability (CIN) constitutes a major contributor to the presence of cytoplasmic DNA [11].

The occurrence of anomalous cytoplasmic dsDNA serves as damage-related molecular patterns (DAMP) that can be detected by the DNA sensor known as cyclic GMP-AMP synthase (cGAS) within the cytoplasm. The binding of cGAS to dsDNA induces an allosteric effect, facilitating the catalysis of cyclic-GMP-AMP (cGAMP) synthesis. cGAMP functions as a second messenger and robust agonist of stimulator of interferon genes (STING). The interaction between cGAMP and STING stimulates the production of type-I interferons (IFN) and initiates the immune responses of the host [12]. Inflammation exerts significant influence as a potent promoter of tumorigenesis [13–15]. The cGAS-STING pathway serves as a mediator of the transcriptional activity encompassing a wide range of molecular processes, including inflammation and the oncogenic progression facilitated by cytokines, chemokines, and growth factors, which promote cellular proliferation, survival, and angiogenesis [16]. The DNA damage response (DDR) acts as a protective mechanism against the replication of DNA lesions by activating multiple cellular events, thereby playing a crucial role in maintaining genomic integrity [17]. CIN stands as a hallmark of human cancer and is closely associated with tumor advancement, therapeutic resistance, and distant metastasis. Cancer cells harboring unstable genomes are susceptible to chromosome missegregation during mitosis, resulting in the formation of micronuclei. These micronuclei possess fragile nuclear envelopes, leading to the exposure of genomic contents to the cytoplasm and subsequently triggering the cGAS-STING pathway, culminating in the production of IFN and inflammatory cytokines [18–20]. According to reports, CIN displays a strong correlation with the progression and metastatic burden observed in PCa [21]. Metastatic and castration-resistant PCa cases featuring defects in DDR showcase characteristics of genomic instability, which significantly impact the prognosis of PCa patients [22].

Considering the age-related nature of PCa and the involvement of the cGAS-STING pathway in both aging and cancer, as indicated by our previous study [23], we have conducted an analysis of molecular subtypes and identified key genes for PCa through the lens of the cGAS-STING pathway. This effort aims to provide a roadmap for the advancement of precision medicine in PCa. Furthermore, we have developed an independent genetic prognosis index to quantitatively assess the risk of recurrence in PCa patients.

## Methods

### Data preparation

We integrated 29 key genes involved in cGAS-STING pathway from previous literatures [11, 24–29]. The PCa gene matrix were obtained from our previous study [30]. Differentially expressed genes and biochemical recurrence (BCR)-free survival-related genes were analyzed. Differential expression was defined based on an absolute log fold change value greater than 0.4 and a *P*_adj_ value smaller than 0.05. Subsequently, we performed an intersection analysis of differentially expressed genes with BCR-related and cGAS-STING-related genes. This allowed us to identify a specific set of genes for grouping 430 PCa patients who underwent radical prostatectomy in the TCGA database using the nonnegative matrix factorization (NMF) algorithm. Furthermore, we established a related risk score based on these genes. MSKCC2010 cohort [31] containing 140 PCa patients undergoing radical prostatectomy were used to externally validated the molecular subtypes and risk score. The prognosis and clinical traits of molecular subtypes were analyzed.

### Mutation landscape and functional differences between two subtypes

RNA-sequencing profiles, genetic mutation data, and corresponding clinical information for PCa were obtained from the TCGA database (https://portal.gdc.com). The maftools package in R software was utilized to download and visualize mutation data. Furthermore, a comparison of mutation frequencies between two subtypes was conducted. The baseline features of two TCGA subtypes in prostate cancer patients was shown in Table 1.

**Table 1:** The baseline features of two TCGA subtypes in prostate cancer patients

Features	Subtype 2	Subtype 1	*P* value
Sample	364	66	
Age, median (IQR)	61 (56, 66)	62 (57.25, 66)	0.247
Gleason score, n (%)			0.255
6	33 (7.7%)	6 (1.4%)	
7	179 (41.6%)	27 (6.3%)	
8	52 (12.1%)	7 (1.6%)	
9	100 (23.3%)	26 (6%)	
T stage, n (%)			0.957
T2	132 (31.1%)	23 (5.4%)	
T3	220 (51.9%)	41 (9.7%)	
T4	7 (1.7%)	1 (0.2%)	
Race, n (%)			0.546
Asian	9 (2.2%)	2 (0.5%)	
Black or African American	45 (10.8%)	5 (1.2%)	
White	297 (71.4%)	58 (13.9%)	
N stage, n (%)			0.949
N0	258 (68.8%)	48 (12.8%)	
N1	59 (15.7%)	10 (2.7%)	
Residual tumor, n (%)			0.231
No	236 (56.3%)	37 (8.8%)	
Yes	119 (28.4%)	27 (6.4%)	

*IQR* interquartile range

Functional analysis involved gene set enrichment analysis utilizing the "c2.cp.kegg.v7.4.symbols.gmt" and "h.all.v7.4.symbols.gmt" datasets from the molecular signatures database [32–34]. Gene set enrichment analysis was performed based on gene expression and subtypes, with a minimum gene set defined as 5 and a maximum gene set defined as 5000. Resampling was conducted 1000 times. A *p*-value of < 0.05 and a false discovery rate of < 0.10 were considered statistically significant. Gene set variation analysis employed a minimum and maximum gene set of 5 and 5000, respectively. The "wilcox.test" function was used to assess the differences in each pathway between the two clusters. A log fold change of 0.4 was considered, and statistical significance was defined as a *P*_adj_ value < 0.01 and a false discovery rate < 0.01.

### Tumor stemness and heterogeneity analyses

The tumor stemness indexes comprised various scores, namely differentially methylated probes-based stemness scores (DMPss), DNA methylation-based stemness scores (DNAss), enhancer elements/DNA methylation-based stemness scores (ENHss), epigenetically regulated DNA methylation-based stemness scores (EREG-METHss), epigenetically regulated RNA expression-based stemness scores (EREG.EXPss), RNA expression-based stemness scores (RNAss), and mRNAsi [35, 36]. Tumor heterogeneity was assessed based on factors such as homologous recombination deficiency (HRD), loss of heterozygosity (LOH), neoantigen (NEO) burden, tumor ploidy, tumor purity, mutant-allele tumor heterogeneity (MATH), tumor mutation burden (TMB), and microsatellite instability (MSI) [37, 38]. The results of the aforementioned indicators were obtained from our previous study [39]. To compare the differences between the two subtypes, the Wilcoxon rank sum test was employed.

### Tumor microenvironment assessment

**Fig. 1:**
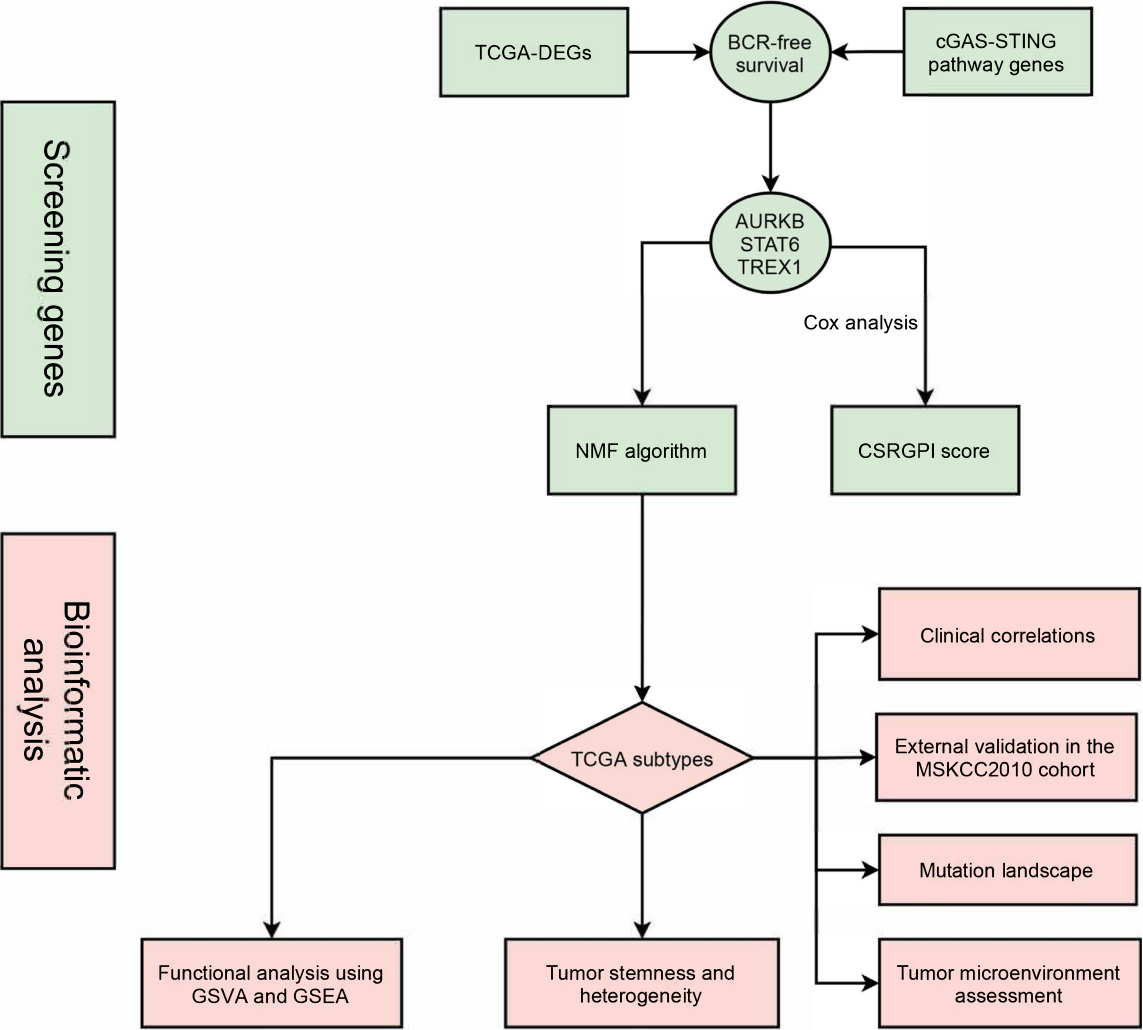
The flowchart of this study. TCGA: the cancer genome atlas; DEGs: differentially expressed genes; BCR: biochemical recurrence; cGAS: cyclic GMP-AMP synthase; STING: stimulator of interferon genes; NMF: nonnegative matrix factorization; CSRGPI: cGAS-STING related gene prognostic index; GSVA: gene set variation analysis; GSEA: gene set enrichment analysis

The comprehensive evaluation of the tumor microenvironment and immune components was conducted using the MCPCounter and ESTIMATE algorithms [40]. The TIDE algorithm was employed to predict the potential response to immune checkpoint blockade (ICB) therapy [41]. A high TIDE score indicates a lower efficacy of ICB. The differences in the expression levels of 54 immune checkpoints and the tumor microenvironment scores between the two subtypes were analyzed using the Wilcoxon rank sum test. Figure 1 illustrates the flowchart outlining the methodology employed in this study.

### Statistical analysis

Analyses were performed using R software version 3.6.3 and relevant packages. The Wilcoxon test was employed for comparisons involving abnormal distributions. Survival analysis was conducted using the log-rank test, and the results were presented as Kaplan–Meier curves. Statistical significance was defined as a two-sided *p*-value of less than 0.05. The notation for indicating significance levels was as follows: not significance (ns), *p* ≥ 0.05; *, *p* < 0.05; **, *p *< 0.01; ***, *p* < 0.001.

## Results

### Identification of cGAS-STING-mediated molecular subtypes and key genes

**Fig. 2:**
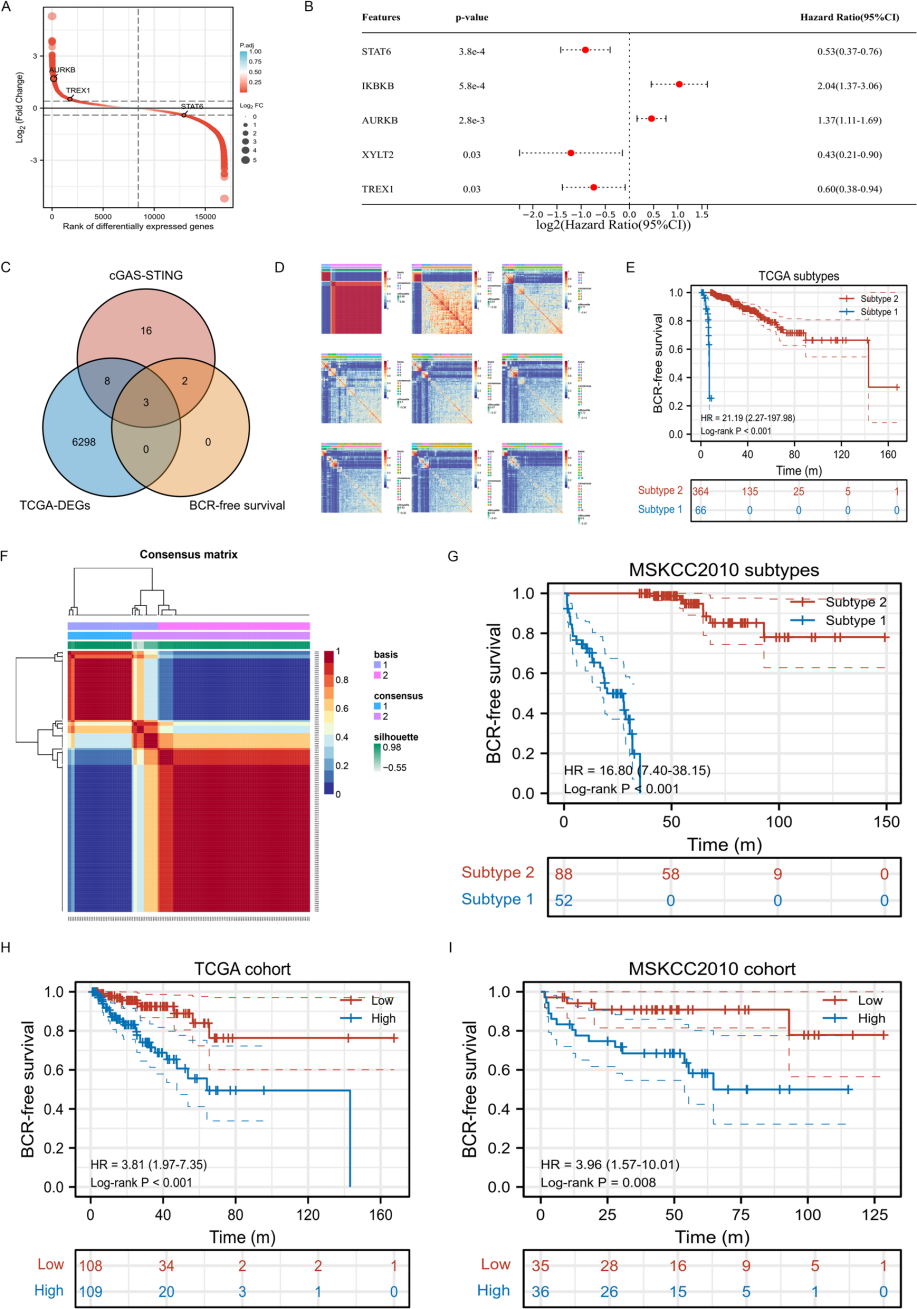
Identification of cGAS-STING-mediated molecular subtypes and key genes (**A**) rank of differentially expressed genes in TCGA database; (**B**) prognosis of differentially expressed genes in TCGA database; (**C**) Venn plot showing intersection of differentially expressed, BCR-free survival-related and cGAS-STING genes; (**D**) heatmap showing all subtypes in TCGA databases; (**E**) Kaplan–Meier curve showing survival differences of TCGA subtypes; (**F**) heatmap showing two subtypes in MSKCC2010 cohort; (**G**) Kaplan–Meier curve showing survival differences of MSKCC2010 cohort; (**H**) Kaplan–Meier curve showing survival differences of high- and low- risk groups in TCGA database; (I) Kaplan–Meier curve showing survival differences of high- and low- risk groups in MSKCC2010 cohort. TCGA: the cancer genome atlas; DEGs: differentially expressed genes; BCR: biochemical recurrence; cGAS: cyclic GMP-AMP synthase; STING: stimulator of interferon genes

Through gene differential expression analysis, we found that three genes including AURKB, TREX1, and STAT6 were with statistical difference (Fig. 2A). Prognosis analyses were performed, which showed different clinical implication among these genes. To be specific, STAT6 (HR:0.53, 95%CI:0.37–0.76), XYLT2 (HR:0.43, 95%CI:0.21–0.90), and TREX1 (HR:0.60, 95%CI:0.38–0.94) were linked to favorable prognosis, but IKBKB (HR:2.04, 95%CI:1.37–3.06) and AURKB (HR:1.37, 95%CI:1.11–1.69) were associated with poor prognosis (Fig. 2B). The differentially expressed genes in TCGA database (TCGA-DEGs) had intersection with cGAS-STING genes and BCR-free survival-related genes. There were 11 genes involving in both cGAS-STING pathway and TCGA-DEGs. There were 5 genes having relationship with both cGAS-STING pathway and BCR-free survival. Thereinto, 3 genes were associated with cGAS-STING pathway, TCGA-DEGs, and BCR-free survival (Fig. 2C). Thus, we conducted subsequent classification analysis using AURKB, TREX1, and STAT6. These genes demonstrated the ability to distinctly classify 430 PCa patients in the TCGA database into two subtypes (Fig. 2D), where subtype 1 had worse prognosis than subtype 2 (HR: 21.19, *p* < 0.001; Fig. 2E). We further validated our findings using MSKCC2010 cohort and similar results were observed (Fig. 2F-G). Using multivariate Cox analysis, we established cGAS-STING related gene prognostic index (CSRGPI). [CSRGPI = -0.38327171969959*STAT6 + 0.341526882562623*AURKB-0.641776317397796*TREX1.] Based on the interquartile range, PCa patients were stratified into high- and low-risk groups. Analysis of both cohorts revealed a significantly elevated risk of BCR in the high-risk group compared to the low-risk group (Fig. 2H-I).

### Mutation landscape and functional analysis

**Fig. 3:**
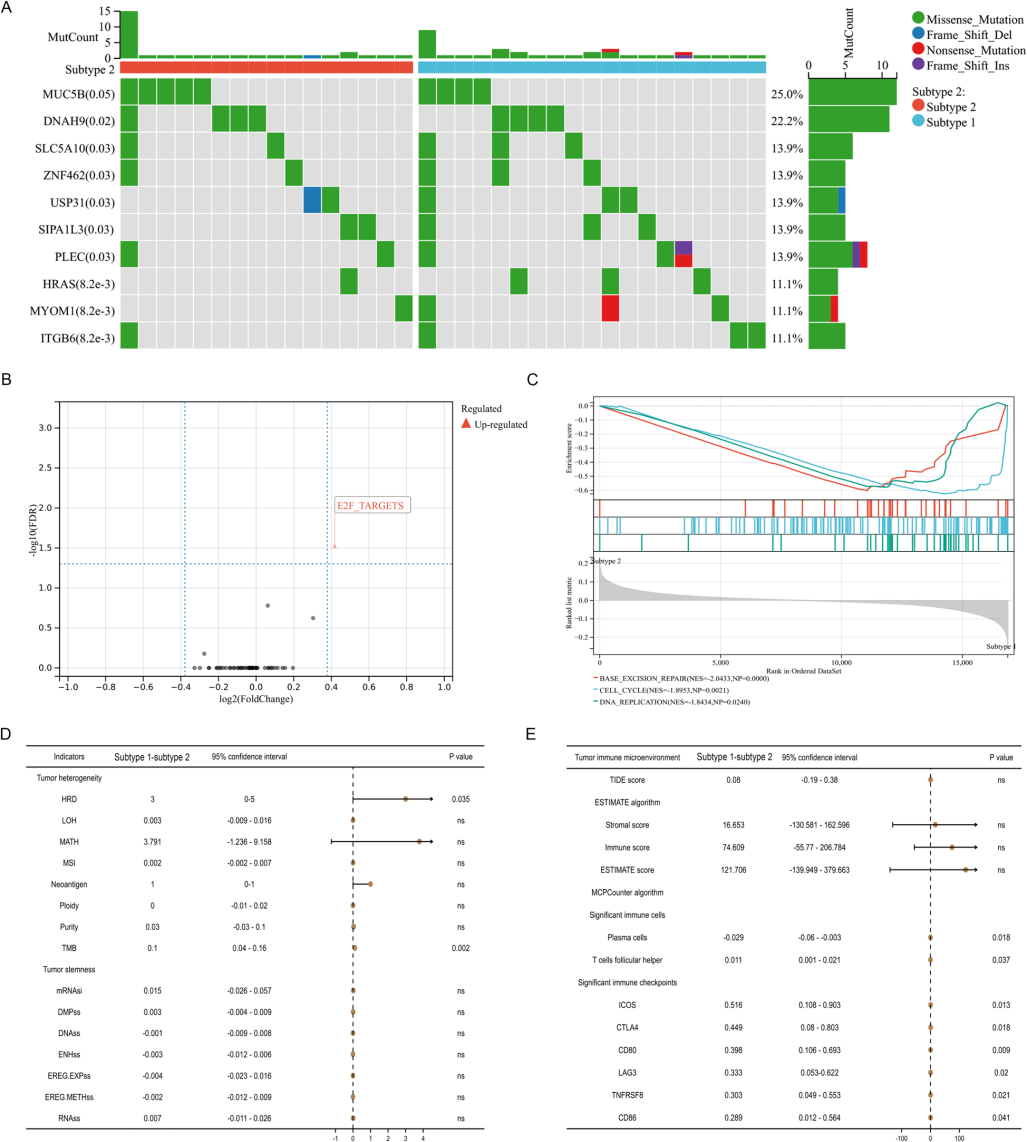
Mutation landscape, functional differences, tumor heterogeneity and stemness and TME between two subtypes. **A **the top ten mutation genes between two subtypes in TCGA database; (**B**) gene set variation analysis; (**C**) gene set enrichment analysis; (**D**) comparison of tumor heterogeneity and stemness between two subtypes in TCGA database; (**E**) comparison of TME and immune checkpoints between two subtypes in TCGA database. TCGA: the cancer genome atlas; TME: tumor immune microenvironment

Between subtype 1 and subtype 2, the top ten mutation genes were MUC5B, DNAH9, SLC5A10, ZNF462, USP31, SIPA1L3, PLEC, HRAS, MYOM1 and ITGB6 (Fig. 3A). Gene set variation analysis (GSVA) revealed a significant enrichment of E2F target genes in subtype 1 (Fig. 3B) and it exhibited a substantial enrichment of base excision repair, cell cycle, and DNA replication, as revealed by gene set enrichment analysis (GSEA) (Fig. 3C).

### Tumor heterogeneity and stemness and tumor immune microenvironment (TME)

In terms of tumor heterogeneity, subtype 1 had significantly higher levels of HRD and TMB than subtype 2. However, there was no significant difference on levels of LOH, MATH, MSI, Neoantigen, Ploidy, Purity among these two subtypes (Fig. 3D). Likewise, we did not identify obvious distinctions in the indicators of tumor stemness between subtype 1 and subtype 2, such as mRNAsi, DMPss, DNAss, ENHss, et al. (Fig. 3D). For TME evaluation, there was no significant difference on TIDE score and ESTIMATE algorithm (including stromal score, immune score, and ESTIMATE score) (Fig. 3E). However, for the levels of significant immune cells, subtype 1 demonstrated a notable elevation in T cells follicular helper and a decrease in plasma cells compared to subtype 2 (Fig. 3E). Regarding the levels of significant immune checkpoints, subtype 1 exhibited significantly higher levels of ICOS, CTLA4, CD80, LAG3, TNFRSF8, and CD86 compared to subtype 2 (Fig. 3E).

## Discussion

There is an exponential increase in the prevalence of PCa due to the fact that approximately additional 1.4 million people had PCa and 375,000 people died for the disease worldwide in 2020 [1]. Although the major determining factors of PCa have not been described, the fact that age and family history are the major determinants has been identified so far [42].

Aging encompasses a temporal progression of functional decline characterized by the gradual deterioration of physiological integrity. This process gives rise to various abnormal human pathologies, including age-related diseases such as cancer, neurodegenerative disorders, diabetes, and cardiovascular ailments. Under physiological circumstances, DNA remains confined within the nucleus and mitochondria, while cytosolic and endolysosomal compartments harbor nucleases capable of DNA degradation. Genomic Instability and mitochondrial dysfunction are two phenotypes of aging, which are the major causes of aberrant release of dsDNA to cytoplasm with age [5].

Endogenous cytoplasmic dsDNA is a major driver of sterile inflammation, also called ‘‘inflamm-aging’’ that regarded as a hallmark of the aging process[43]. Genomic instability is associated with the formation of micronuclei. Micronuclei are small membrane-bounded compartments encapsulated by a nuclear envelope with chromosomes or chromosome fragments. Nevertheless, the nuclear envelope of micronuclei exhibits structural deficiencies, rendering it susceptible to fragmentation upon exposure to both internal and external stressors. Consequently, the DNA content within these micronuclei can be released directly into the cytoplasm. Mitochondria, on the other hand, consist of two distinct membranes, namely the outer and inner membranes, which play vital roles in facilitating intracellular metabolism and energy production. Within the inner mitochondrial matrix, discrete nucleoids organize the mtDNA [44, 45].

Age-related mitochondrial dysfunction is marked by reduced respiratory capacity per individual mitochondrion, accompanied by diminished mitochondrial membrane potential and an associated increase in the generation of reactive oxygen species (ROS). This impairment of mitochondrial function acts as both a cause and a consequence of cellular senescence, prompting the permeabilization of mitochondrial membranes and initiating either apoptosis or necrosis [46, 47]. The proapoptotic proteins BAX/BAK are expressed on the outer mitochondrial membrane, where they assemble into macropores, leading to the induction of mitochondrial outer membrane permeabilization (MOMP) upon sensing apoptotic signals [48]. Following the activation of BAK/BAX and subsequent loss of cytochrome c, the integrity of the mitochondrial network becomes compromised. Additionally, the pores formed by BAX/BAK on the outer mitochondrial membrane gradually expand, leading to the protrusion of the inner mitochondrial membrane, which contains the mtDNA, into the cytoplasm [49]. cGAS is a protein consisting of 520 amino acids that typically remains in an inactive state. It comprises an unstructured and highly basic N-terminus, spanning 160 amino acids, and a globular structural domain composed of 360 amino acids [12].

Recent investigations have revealed that cGAS is not solely confined to the cytoplasm, but also exhibits constitutive and predominant localization within the nucleus. Despite its role as a DNA sensor and interaction with nuclear DNA, the catalytic function of cGAS is impeded due to its strong tethering to the nucleus. This tethering occurs as cGAS binds to a negatively charged acidic region created by histones, utilizing its second DNA-binding site. The high-affinity binding to nucleosomes obstructs the binding of double-stranded DNA and consequently induces the inactivation of cGAS [50]. Nonetheless, sequestering cGAS in an inactive state serves to maintain a low level of soluble cGAS. Upon stimulation by agonistic double-stranded DNA (dsDNA), the high-affinity nucleosome-bound cGAS swiftly replenishes the soluble cGAS pool without requiring de novo protein synthesis. This mechanism plays a crucial role in achieving an optimal dynamic range and facilitating a rapid response in scenarios where soluble cGAS becomes depleted due to the excessive presence of aberrant dsDNA leakage in the cytoplasm [51]. Upon encountering dsDNA, cytoplasmic cGAS undergoes a conformational change, leading to its activation and the subsequent generation of the second messenger molecule, cGAMP. Subsequently, cGAMP binds to STING, which is situated within the endoplasmic reticulum (ER). This binding event triggers a reconfiguration of STING's conformation, facilitating its translocation from the ER to the Golgi apparatus via the ER-Golgi intermediate compartment (ERGIC). Within the Golgi, STING undergoes polymerization and activates TANK-binding kinase 1 (TBK1) [24, 52]. Following activation, TBK1 phosphorylates STING, leading to the recruitment of interferon regulatory factor 3 (IRF3). Subsequently, TBK1 further phosphorylates IRF3, prompting its dimerization and subsequent translocation into the nucleus. This cascade of events initiates the production of interferons (IFNs) and other target genes, including those encoding inflammatory cytokines such as IL6 and IL12. The transcriptional response induced by IRF3 varies across different cell types and depends on the specific triggering of STING as well as the precise in vivo environment [10].

The cGAS-STING pathway has emerged as a significant mechanism involved in inflammation-driven tumorigenesis. The pathway exerts its effects through downstream effector molecules, including TBK1, which has been associated with sustained inflammation and the progression of cancer [53, 54]. Considering the significant involvement of the cGAS-STING pathway in the processes of aging and inflammation, it is plausible to establish a connection between cGAS-STING and age-related diseases as well as inflammation-associated tumors. PCa stands as a notable example of such diseases [55, 56].

Our findings provide evidence that AURKB, STAT6, and TREX1 are among the genes associated with the cGAS-STING pathway, contributing to the BCR-free survival of PCa patients. AURKB, belonging to the Aurora kinase gene family, is a highly conserved gene involved in threonine/serine regulation in mammals. It forms the chromosomal passenger complex (CPC) along with proteins such as Survivin, Borealin, and the Inner Centromere Protein. Throughout mitosis, the CPC dynamically localizes to different regions of the centromere, including the prophase and metaphase centromeres, the cortex and spindle midzone during anaphase, and the mid-body during telophase. Its crucial functions encompass chromosome segregation and cytokinesis processes [57, 58].

A protein complex consisting of AURKB and P53 has been identified, wherein AURKB phosphorylates serine and threonine residues at position 284 of P53. Perturbations in AURKB expression result in inhibiting the transcriptional activity of p53, thereby compromising its role as a tumor suppressor [59]. AURKB exhibits high expression levels in primary human prostate cancer (PCa) and its cell lines. Nuclear localization of AURKB in prostatic intraepithelial neoplasia lesions is associated with clinical staging [60, 61]. Additionally, elevated levels of transforming growth factor β (TGFβ) have been observed in certain advanced cancers, contributing to tumor progression. AURKB can form a complex with TGFβ receptor (TβR), known as the AURKB-TβRI complex, which correlates with the malignancy of PCa and serves as a potential prognostic biomarker for patients at risk of developing aggressive PCa [62]. Addepalli et al. demonstrated that the knockdown of AURKB through RNAi inhibited PCa growth in athymic nude mice [63]. STAT6 is a transcription factor involved in tumorigenesis and the regulation of the tumor microenvironment. Its activation depends on the phosphorylation of conserved tyrosine residues on the receptor by cytokines. Phosphorylated STAT6 forms homodimers and translocates to the nucleus, where its DNA-binding domain recognizes and binds to specific DNA sequences, activating the transcription and translation of target genes [64]. Das et al. examined the expression of STAT6 in clinical PCa tissue specimens and found its presence in prostate intraepithelial neoplasia, malignant epithelial layers, and particularly high expression in the fibromuscular stroma. Furthermore, the expression level of STAT6 was significantly associated with tumor size and high histological grades of PCa [65]. TREX1 is a 3'-5' DNA exonuclease associated with the ER. Following the rupture of the micronuclear envelope, TREX1 translocates into the nucleus and degrades micronuclear DNA. This process leads to a decrease in cytoplasmic DNA levels and the inhibition of activation of the cGAS-STING pathway [66]. Moreover, TREX1 is capable of degrading tumor-derived DNA in the cytoplasm, thereby compromising the IFN-dependent antitumor immunity induced by the cGAS-STING pathway. Consequently, inhibition of TREX1 exonuclease activity is considered a potential immunotherapeutic strategy to restore antitumor immunity by reducing the degradation of tumor-derived DNA [67]. Interestingly, cancer cells exhibit cytoplasmic accumulation of DNA, which is associated with the limited activity of TREX1 induced by various mechanisms in cancers. This accumulation leads to chronic inflammation and the suppression of an effective immune response [68]. Additionally, the downregulation of TREX1 expression in senescent cells may contribute to the aberrant activation of the cGAS-STING pathway and the accumulation of cytoplasmic DNA observed in cancers [69].

Considering the strong association between aging and the cGAS-STING pathway in PCa, we identified key genes involved in this pathway and computed a gene prognostic index comprising AURKB, STAT6, and TREX1. Using the NMF algorithm as described earlier, we stratified PCa patients into two subtypes. Subtype 1 exhibited a significantly higher risk of BCR compared to subtype 2. Additionally, we developed a risk score to quantify the BCR risk in PCa patients, and the high-risk group showed a significantly greater BCR risk than the low-risk group. Analysis of mutation genes between subtype 1 and subtype 2 revealed several noteworthy findings. MUC5B was associated with the acquisition of hormone independence and preferentially expressed in hormone-independent PCa [70]. PLEC played a crucial role as a regulator in PCa development and metastasis [71]. ITGB6 encoded the β6 integrin subunit, with significantly higher expression observed in metastatic castrate-resistant androgen receptor-negative prostate tumors compared to androgen receptor-positive tumors [72]. Approximately 10% of CRPC patients exhibited HRD, and patients with M1 stage, high Gleason score, and IDC-P pathology demonstrated higher HRD scores, which correlated with poor prognosis in PCa [73, 74]. Consistently, we observed that subtype 1, associated with worse prognosis, exhibited a significantly higher HRD score than subtype 2. Furthermore, patients in subtype 1 might display sensitivity to immune therapy, as they exhibited a significantly higher TMB than subtype 2.

The limitations of our study must be mentioned. First, we did not conduct experimental validations following our analyses, which may hamper the clinical transformation of our findings. Further in-depth researches are promptly warranted. Second, besides BCR-free survival, other outcomes such as overall survival or cancer-specific survival deserve to be carefully considered as well. Third, due to the complexity of molecular pathways in tumor biology, the interaction between cGAS-STING and others may cause undetected influence on PCa, making it difficult to elucidate the detailed impacts induced by a single mechanism.

The global population is experiencing rapid aging, presenting numerous challenges. Among these challenges is the increased morbidity of PCa, which disproportionately affects older males and leads to disability, diminished quality of life, escalated costs, and even mortality. Consequently, it becomes crucial to carefully select PCa patients and identify those who are at a high risk of BCR and disease progression. Our study results have provided sufficient evidence that the prognostic index is a reliable predictor of BCR-free survival in individual PCa patients.

## Conclusions

Our study revealed that the utilization of molecular subtypes and genetic risk score that associated with the cGAS-STING pathway could assist clinicians in identifying PCa patients who harbor unfavorable tumor feature. This identification enables the selection of appropriate pharmacologic therapies that specifically target the cGAS-STING pathway, thereby maximizing the potential benefits for these patients.

## Data Availability

The datasets generated and/or analyzed during the current study are available in the TCGA (https://www.cancer.gov/tcga) and GEO (https://www.ncbi.nlm.nih.gov/geo/, accession number: GSE116918 and GSE46602) repositories. All data can be accessible from corresponding authors with reasonable requests.

## Abbreviations

PCa: Prostate cancer
cGAS: Cyclic GMP-AMP synthase
cGAMP: Cyclic-GMP-AMP
STING: Stimulator of interferon genes
TCGA: The cancer genome atlas
CSRGPI: CGAS-STING related gene prognostic index
DNA: Deoxyribonucleic acid
mtDNA: Mitochondrial DNA
VDAC: Voltage- dependent anion channel
MPTP: Mitochondrial permeability transition pore
dsDNA: Double-stranded DNA
CIN: Chromosomal instability
DAMP: Damage-related molecular patterns
IFN: Type-I interferons
DDR: DNA damage response
BCR: Biochemical recurrence
NMF: Nonnegative matrix factorization
DMPss: Differentially methylated probes-based stemness scores
DNAss: DNA methylation-based stemness scores
ENHss: Enhancer elements/DNA methylation-based stemness scores
EREG-METHss: Epigenetically regulated DNA methylation-based stemness scores
EREG.EXPss: Epigenetically regulated RNA expression-based stemness scores
RNAss: RNA expression-based stemness scores
HRD: Homologous recombination deficiency
LOH: Loss of heterozygosity
NEO: Neoantigen
MATH: Mutant-allele tumor heterogeneity
TMB: Tumor mutation burden
MSI: Microsatellite instability
ICB: Immune checkpoint blockade
ROS: Reactive oxygen species
MOMP: Mitochondrial outer-membrane permeabilization
ER: Endoplasmic reticulum
ERGIC: ER-Golgi intermediate compartment
IRF3: Interferon regulatory factor 3
CPC: Chromosomal passenger complex
TGFβ: Transforming growth factor β
GSVA: Gene set variation analysis
GSEA: Gene set enrichment analysis
